# Evaluating the evaluators: does C-SATS measure up?

**DOI:** 10.1007/s00464-025-12150-0

**Published:** 2025-09-15

**Authors:** Robert B. Laverty, Charles H. Chesnut, Joseph R. Karam, Joseph C. L’Huillier, Alexander Bonte, Julie M. Clanahan, Jisuk Park, Brian Yoon, Robert W. Krell

**Affiliations:** 1 Department of Surgery, Brooke Army Medical Center, San Antonio, TX USA; 2 General Surgery Residency–Valley System Health, Las Vegas, NV USA; 3 Start Center for Surgical Care, San Antonio, TX USA; 4 Department of Surgery, University at Buffalo, Buffalo, NY USA; 5 Department of Surgery, Hackensack University Medical School, Hackensack, NJ USA; 6 Department of Surgery, Washington University in St. Louis, St. Louis, MO USA; 7 Science and Technology, 59th Medical Wing, Lackland AFB, TX USA; 8 Evans Army Community Hospital, Fort Carson, CO USA

**Keywords:** Robotic surgery, General surgery, Surgical competency

## Abstract

**Introduction:**

Robotic-assisted surgery has increased in prevalence, particularly in general surgery. The number of cases required to achieve adequate proficiency in robotic surgery, however, and the training metrics that correlate best with proficiency remain unclear. We sought to better define proficiency-based benchmarks in robotic-assisted cholecystectomies (RAC) and inguinal hernia repairs (RIHR) using a commercial crowd source based on competency platform.

**Methods:**

Multi-institutional cohort study in which 48 surgeons (senior residents, fellows, and practicing physicians) submitted representative videos of themselves performing a RAC and/or RIHR. Subjects subsequently underwent blinded case video reviews using the C-SATS platform, which utilizes the Global Evaluative Assessment of Robotic Skills (GEARS) rubric. Participating surgeons self-reported surgical case volume. Primary outcome was correlation of GEARS scores with historic procedure case volume. Secondary outcomes included construct validity of GEARS scores as an operative proficiency metric.

**Results:**

Total GEARS scores and historical case volume showed positive correlation for both RAC (*r* = 0.65, *p* < 0.0001) and RIHR (*r* = 0.54, *p* = 0.001) among all performers. On subgroup analysis, no correlation was seen for resident/fellow physicians (*r* = 0.39, *p* = 0.11 for RAC; *r* = 0.22, *p* = 0.49 for RIHR) or those with < 50 historic case volume (*r* = 0.14, *p* = 0.55 for RAC; *r *= 0.21, *p* = 0.54 for RIHR). No difference in total GEARS scores was seen between resident/fellow and practicing physicians for either RAC (20.21 v 20.25, *p* = 0.82) or RIHR (20.45 v 20.46, *p* = 0.95), nor in those with < 50 or ≥ 50 historic case volume in RAC (20.16 v 20.33, *p* = 0.33) and RIHR (20.35 v 20.49, *p* = 0.48). GEARS scores by domain (bimanual dexterity, depth perception, efficiency, force sensitivity, and robotic control) and surgical step (exposure of triangle of calot, clipping and division of cystic artery/duct, and dissection of gallbladder; mobilizing peritoneal flap, hernia sac dissection, and mesh placement) were similar across both groups (*p* > 0.05).

**Conclusion:**

C-SATS-derived GEARS scores correlated to overall surgeon historical case volume for RA cholecystectomy and IHR, but not among novice performers. This methodology was unable to differentiate between novice and expert performers for these procedures. There remains a need for high-fidelity and discerning robotic skills evaluation platforms for trainees and novice surgeons.

**Graphical abstract:**

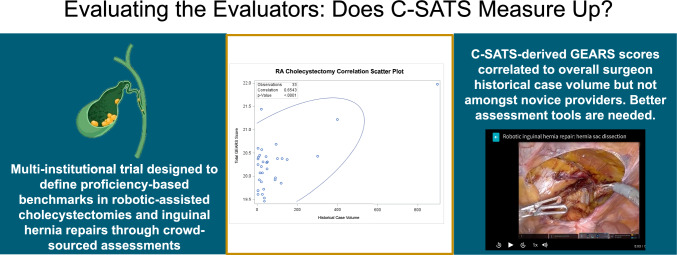

Robotic-assisted surgery has increased in prevalence, particularly in general surgery, since initial FDA approval in 2000. While many surgeons cite the platform’s visual and ergonomic advantages compared to laparoscopy, high-profile complications have led to increased public scrutiny regarding proper use of the technology [1, 2]. Two particular concerns are the extent to which robotic surgeons are trained in the platform’s use and the variability in hospital credentialing processes [3]. Given the correlation between surgeon technical skill and patient outcomes, determining proficiency-based metrics for competence in videoscopic surgery is of the utmost importance [4–7]. Such metrics exist for laparoscopy, in which didactic and skills-based testing is required for board certification in general surgery; however, no such requirements exist for robotic-assisted surgery. For example, guidance from the Society of American Gastrointestinal and Endoscopic Surgeons regarding robotic surgical privileges does not cite specific metrics, instead stating independent practice should be based on an “appropriate volume of cases.”

The number of cases required to achieve adequate proficiency in robotic surgery and the training metrics that correlate best with proficiency remain unclear. Published studies concluded anywhere from 10 to 50 cases were required for appropriate skills acquisition in robotic-assisted inguinal hernia repair and cholecystectomy [8, 9]. However, these studies used operative time as their basis for proficiency, which has been demonstrated to show no correlation with standardized assessments of technical skill [10, 11]. Recently, work has been done to develop video-based assessments for such a purpose using expert evaluators; however, these efforts are unlikely to be able to scale in order to meet the worldwide demand for qualification and credentialing [12]. We sought to define proficiency-based competency metrics in commonly performed robotic general surgery procedures—robotic-assisted inguinal hernia repair (RIHR) and cholecystectomy (RAC)—using the Crowd-Sourced Assessment of Technical Skill (C-SATS) platform, which uses the Global Evaluative Assessment of Robotic Skills (GEARS) rubric. This is a standardized clinical assessment tool for robotic surgical skills, which has been validated in multiple studies [13–15]. The tool assesses 6 separate domains—depth perception, bimanual dexterity, efficiency, autonomy, force sensitivity, and robotic control—each of which is scored using a 5-point Likert-like scale with specific performance anchors at 1, 3, and 5, then averaged across all evaluators. More specifically, we set out to evaluate GEARS scores in RIHR and RAC across a spectrum of surgeon experience levels to, in turn, determine the construct validity of GEARS as a general surgery operative proficiency metric and establish the correlation of GEARS scores with other commonly measured metrics like operative time and historic case volume to determine whether GEARS is a more appropriate metric for credentialing.

## Materials and methods

This was a multi-institutional, cross-sectional study in which senior general surgery residents (PGY-4/5) and surgeons in fellowship or practice submitted videos of themselves performing a RIHR and/or RAC. Participants were recruited via email and other advertising methods. The study received an exempt IRB determination for data collection from all participating institutions. Submitted videos had to be performed by the participant in its entirety and within the year prior to submission. RAC video eligibility criteria were those performed in the elective setting for symptomatic cholelithiasis, biliary dyskinesia, or gallbladder polyps; RIHR criteria included elective repairs for reducible, non-scrotal inguinal hernias. Surgeons then self-reported historical surgical case volume and select demographic information.

The videos were uploaded to the C-SATS platform for blinded review using the Global Evaluative Assessment of Robotic Skills (GEARS) rubric. C-SATS is a company based in Seattle, Washington which has experience performing secure OR video acquisition and GEARS assessments [11]. Their methodology of training non-expert medical personnel to perform these analyses has been validated in a number of different settings [16–19]. Reviewers were not known to the participating surgeons/residents submitting videos, which allowed for an unbiased assessment of surgeon skill. C-SATS was unable to disclose their data regarding inter-rater reliability for these assessments but reported all as being greater than 0.9.

To ensure we obtain a representative cross section of surgeons, we recruited similar numbers of participants according to experience level. Continuous data were assessed using appropriate parametric and non-parametric tests (e.g., t-test or Wilcoxon Rank-Sum test; Pearson correlation or Spearman rank correlation); categorical data with chi-squared tests. For all statistical tests, p values are two-tailed, and alpha is set at 0.05, with no correction for multiple comparisons. Analyses were performed using Statistical Analysis Software version 9.4 (Cary, NC).

## Results

**Fig. 1:**
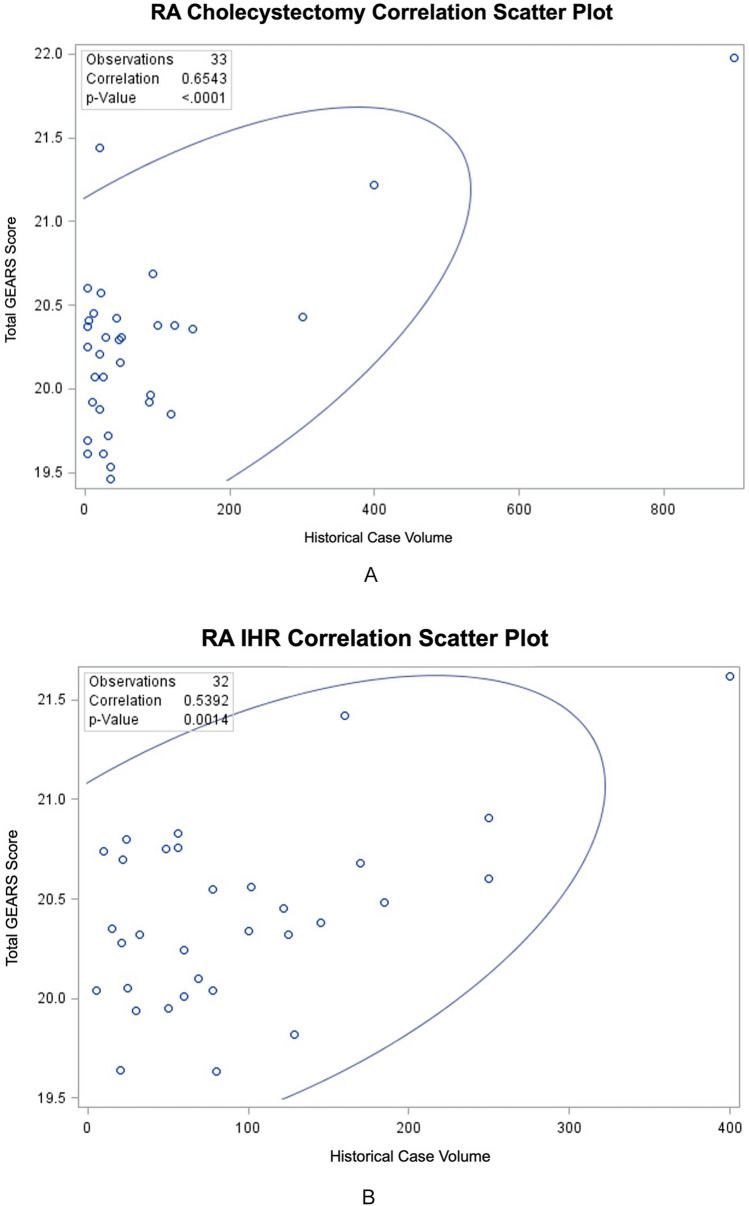
**A** Correlation between total GEARS scores and historical case volume of all surgeons performing RA cholecystectomy, scatter plot with 95% prediction ellipses. **B** Correlation between total GEARS scores and historical case volume of all surgeons performing RA IHR, scatter plot with 95% prediction ellipses

A total of 48 surgeons submitted 70 videos of RIHR and RAC, the demographics of whom can be seen in Table 1. Significant correlations were demonstrated between total GEARS scores and historical case volume for both RA cholecystectomy (*r* = 0.65, *p* < 0.0001) and IHR (*r* = 0.54, *p* = 0.001) among all performers (Fig. 1A, B). The statistical significance of the association was driven by practicing surgeons with > 50 cases (*r* = 0.90, *p* = 0.0004 for RAC; *r* = 0.68, *p* = 0.003 for RIHR). In contrast, statistical significance was not achieved for correlations among resident/fellow physicians (*r* = 0.39, *p* = 0.11 for RAC; *r* = 0.22, *p* = 0.49 for RIHR) or those with < 50 historic case volume (*r* = 0.14, *p* = 0.55 for RAC; *r* = 0.21, *p* = 0.54 for RIHR). The correlation among total GEARS scores and historic case volume among practicing surgeons was significant (*r* = 0.90, *p* = 0.0004 for RAC; *r* = 0.68, *p* = 0.003 for RIHR).

**Table 1:** Surgeon demographic information, historic case volume, and case times

Variable	RA chole (*n* = 37)	RA IHR (*n* = 33)
Age, median (IQR)	35 (8.5)	35 (8.25)
Male, *n* (%)	31 (84)	25 (76)
Resident/Fellow, *n* (%)	20 (54)	15 (45)
Formal robotic curriculum completed, *n* (%)	30 (81)	23 (76)
Historic volume of IHR/Chole		
Total open, mean (SD)	19 (73)	83 (205)
Total laparoscopic, mean (SD)	149 (200)	29 (36)
Total robotic, mean (SD)	71 (146)	103 (93)
Volume of IHR/Chole in past year		
Open, mean (SD)	2 (5)	6 (5)
Laparoscopic, mean (SD)	28 (41)	3 (4)
Robotic, mean (SD)	27 (34)	38 (31)
Case duration (minutes)		
Residents/fellows, median (IQR)	36 (31)	50 (48.5)
Practicing surgeons, median (IQR)	32 (13)	53.5 (41.5)
Case volume < 50, median (IQR)	34.5 (16)	52 (59)
Case volume ≥ 50, median (IQR)	31 (31)	48 (32)

Case duration was found to be similar across all groups for both RAC and RIHR (Table 1, *p* > 0.05). No significant negative correlation was demonstrated among all participants between case duration and total GEARS scores (*r* = − 0.29, *p* = 0.09 for RAC; *r* = − 0.33, *p* = 0.06 for RIHR). Only significant negative correlations among these groupings between case duration and total GEARS scores are shown in the practicing surgeon group for RAC (*r* = − 0.58, *p* = 0.014), and those with ≥ 50 historic case volume (*r* = − 0.65, *p* = 0.011 for RAC; *r* = − 0.44, *p* = 0.04 for RIHR).

No difference in total GEARS scores was seen between resident/fellow and practicing physicians for either RAC (20.21 v 20.25, *p* = 0.82) or RIHR (20.45 v 20.46, *p* = 0.95), nor in those with < 50 or ≥ 50 historic case volume in RAC (20.16 v 20.33, *p* = 0.33) and RIHR (20.35 v 20.49, *p* = 0.48). GEARS scores by domain (bimanual dexterity, depth perception, efficiency, force sensitivity, and robotic control) and surgical step (exposure of triangle of calot, clipping and division of cystic artery/duct, and dissection of gallbladder; mobilizing peritoneal flap, hernia sac dissection, and mesh placement) were similar across both groups (Tables 2, 3, *p* > 0.05).

**Table 2:** GEARS scores by domain in RA cholecystectomy, all *p* > 0.05

Surgical step	Resident/fellow (*n* = 20)	Practicing surgeon (*n* = 17)
Exposure of the triangle of calot		
Bimanual dexterity, mean (SD)	4.04 (0.15)	3.96 (0.18)
Depth perception, mean (SD)	4.11 (0.16)	4.13 (0.11)
Efficiency, mean (SD)	3.85 (0.22)	3.85 (0.22)
Force sensitivity, mean (SD)	4.13 (0.20)	4.10 (0.20)
Robotic control, mean (SD)	4.15 (0.16)	4.16 (0.22)
Clipping and division of the cystic duct and artery		
Bimanual dexterity, mean (SD)	3.98 (0.17)	3.97 (0.69)
Depth perception, mean (SD)	4.03 (0.13)	4.12 (0.23)
Efficiency, mean (SD)	3.82 (0.20)	3.81 (0.14)
Force sensitivity, mean (SD)	4.17 (0.20)	4.16 (0.24)
Robotic control, mean (SD)	4.18 (0.16)	4.16 (0.23)
Dissection of the gallbladder		
Bimanual dexterity, mean (SD)	4.0 (0.13)	4.02 (0.21)
Depth perception, mean (SD)	4.13 (0.12)	4.11 (0.21)
Efficiency, mean (SD)	3.91 (0.17)	3.83 (0.19)
Force sensitivity, mean (SD)	4.08 (0.25)	4.17 (0.26)
Robotic control, mean (SD)	4.11 (0.15)	4.14 (0.26)

**Table 3:** GEARS scores by domain in RA IHR, all *p* > 0.05

Surgical step	Resident/fellow (*n* = 15)	Practicing surgeon (*n* = 20)
Mobilizing peritoneal flap		
Bimanual dexterity, mean (SD)	4.0 (0.12)	4.04 (0.14)
Depth perception, mean (SD)	4.19 (0.15)	4.12 (0.15)
Efficiency, mean (SD)	3.9 (0.13)	3.92 (0.24)
Force sensitivity, mean (SD)	4.16 (0.10)	4.21 (0.22)
Robotic control, mean (SD)	4.23 (0.10)	4.22 (0.18)
Hernia sac dissection		
Bimanual dexterity, mean (SD)	4.04 (0.15)	4.11 (0.19)
Depth perception, mean (SD)	4.12 (0.12)	4.12 (0.15)
Efficiency, mean (SD)	3.85 (0.19)	3.93 (0.19)
Force sensitivity, mean (SD)	4.07 (0.12)	4.19 (0.24)
Robotic control, mean (SD)	4.22 (0.18)	4.24 (0.18)
Mesh placement		
Bimanual dexterity, mean (SD)	4.09 (0.13)	3.98 (0.16)
Depth perception, mean (SD)	4.16 (0.12)	4.07 (0.22)
Efficiency, mean (SD)	3.86 (0.24)	3.77 (0.21)
Force sensitivity, mean (SD)	4.20 (0.17)	4.22 (0.19)
Robotic control, mean (SD)	4.18 (0.14)	4.11 (0.21)

## Discussion

As aforementioned, GEARS has been validated in a number of clinical settings as a metric for surgical proficiency, but not in the realm of general surgical procedures [13, 15]. When used by crowd-sourced assessors, GEARS did not meet the mark for construct validity in RIHR and RAC. No significant difference was seen between novices and experts in either of these procedures.

A significant correlation was demonstrated between total GEARS scores and historic case volume among all participants; however, that significance was lost when the novices and experts were separated. A similar trend was seen in case duration analyses. Specifically, no correlation was seen among resident and fellow participants for whom potential application of this technology for credentialing and ongoing training would be most useful. C-SATS seemed incapable of distinguishing participants on the early end of the learning curve based upon historic case volume and operative time. And even though significance was reached in the expert population, which likely distorted the result among all participants, the GEARS score range was very narrow, raising questions as to its utility and clinical significance in this cohort as well. Since the standard deviation of these scores for all participants was < 0.5, it would be challenging to determine an inflection point on the GEARS score learning curve at which you could determine competency.

Video-based assessments of technical skill have the potential to facilitate training, improve credentialing, and make surgery safer worldwide. The means by which to evaluate these videos on a large-scale basis, however, remain to be seen. We believe that the most promising solutions lie within the field of artificial intelligence for evaluation of surgical performance. As technology develops further with regard to robotic surgery technology, there will be enhanced integration of artificial intelligence to better assess surgical procedures and outcomes. There will be improvements in technology like real-time ICG fluorescence, integrated radiographic imaging, and haptics to help further reduce injuries and improve outcomes.

Of note, comparing laparoscopic versus robotic-assisted surgery in the recent literature has demonstrated equivalent outcomes with regard to surgical outcomes. One study suggested that there was an increased rate for bile duct injury with robotic-assisted cholecystectomy; however, another study demonstrated a decrease in conversion to open [20, 21]. These new studies highlight the importance of the need for improved training and proficiency benchmarks while learning the robotic surgery technology.

This study has several limitations that warrant consideration. First, the sample size (*n* = 48) limits statistical power, particularly for subgroup analyses. While recruitment efforts were extensive, a substantially larger cohort would be required to detect subtle differences in performance across experience levels. Second, the study’s cross-sectional design precludes longitudinal assessment of skill acquisition over time, which limits our understanding of how GEARS scores evolve with growing experience. Third, the GEARS rubric, although previously validated in other surgical disciplines, demonstrated limited discriminatory power in this study for general surgery procedures, potentially due to ceiling effects or lack of sensitivity to early learning curve nuances. The rubric also does not directly evaluate task-specific elements such as the Critical View of Safety or myopectineal orifice dissection, which may be crucial for accurate skill differentiation in robotic cholecystectomy and hernia repair. Fourth, the lack of correlation between GEARS scores and historical case volumes among novice surgeons suggests that additional objective metrics, such as robotic console data (e.g., path length, error rate), may be necessary to more precisely capture proficiency in early learners. Fifth, although video reviewers were blinded to participant identity, the absence of inter-rater reliability data from the commercial platform (C-SATS) limits transparency in scoring consistency. Finally, the study relied exclusively on a single commercial evaluation platform, which may limit external validity and generalizability. Future work should aim to validate these findings using alternative platforms, incorporate longitudinal designs, and evaluate links between technical assessments and patient outcomes.

The use of robotic-assisted cholecystectomy has increased 37-fold between 2010 and 2019. The overall application of this technology is ever growing, increasing access to minimally invasive surgery care for patients. We need to integrate consistent ways to evaluate surgeons in order to decrease the learning curve and improve patient outcomes. C-SATS did not meet the mark; we need to develop a technology that will.
